# Design and Implementation of Improved SARS-CoV-2 Diagnostic Assays To Mitigate the Impact of Genomic Mutations on Target Failure: the Xpert Xpress SARS-CoV-2 Experience

**DOI:** 10.1128/spectrum.01355-22

**Published:** 2022-10-18

**Authors:** Bethany L. Burns, Domonique Moody, Zheng Jin Tu, Joy Nakitandwe, Jay E. Brock, David Bosler, Stephanie L. Mitchell, Michael J. Loeffelholz, Daniel D. Rhoads

**Affiliations:** a Department of Laboratory Medicine, Cleveland Clinic, Cleveland, Ohio, USA; b Department of Pathology, Cleveland Clinic Lerner College of Medicine, Case Western Reserve University, Cleveland, Ohio, USA; c Cepheid, Sunnyvale, California, USA; d Infection Biology Program, Lerner Research Institute, Cleveland Clinic, Cleveland, Ohio, USA; University of California, San Diego

**Keywords:** Cepheid, FDA emergency use authorization, gene mutations, NGTF, SARS-CoV-2, target failures, Xpert, Xpress

## Abstract

In 2020, the U.S. Food and Drug Administration (FDA) enabled manufacturers to request emergency use authorization (EUA) to facilitate the rapid authorization of *in vitro* diagnostic (IVD) platforms for the detection of severe acute respiratory syndrome coronavirus 2 (SARS-CoV-2). Uncommon SARS-CoV-2 point mutations could cause nucleocapsid (N) gene target failure (NGTF) when using first-generation Xpert Xpress assays, so improvements were designed and implemented. In response to NGTF reports and with consideration of viral genomic information in public databases, the Xpress assays were redesigned to mitigate the impact of SARS-CoV-2 mutations on qualitative assay performance. The second-generation assays include a third gene target (RNA-dependent RNA polymerase [RdRp]) and redundant oligonucleotide probes for the N2 target. First- and second-generation assay performances were evaluated using a challenge set of samples. A second-generation assay with updated oligonucleotide chemistry received FDA EUA in September 2021. A prototype assay with oligonucleotide chemistry similar to that of the second-generation assay with FDA EUA successfully detected all three gene targets (N2, envelope [E], and RdRp) in all challenge samples (100%; 50/50), including variants with N gene mutations (g.29197C>T or g.29200C>T), which caused NGTF in the first-generation assays. Investigation and reporting of IVD target failures, public sharing of viral genomic sequence data, and the FDA EUA pathway were essential components in facilitating a short cycle time from the identification of mutations that impact the performance of an IVD assay to the design and implementation of an improved IVD assay.

**IMPORTANCE** The SARS-CoV-2 genome has mutated during the coronavirus disease 2019 (COVID-19) pandemic. Some of these mutations have impacted the performance of nucleic acid amplification tests like PCR, which are commonly used as diagnostic tools to detect an infection. The U.S. Food and Drug Administration (FDA) emergency use authorization (EUA) process enables the rapid reformulation and regulatory authorization of improved PCRs. In our experience, the identification of SARS-CoV-2 mutations that impact PCR performance, the subsequent development of improved PCR chemistry, and the use of the FDA EUA regulatory pathway led to improved diagnostic performance during the SARS-CoV-2 pandemic that is able to keep pace with the rapidly evolving genome of SARS-CoV-2.

## INTRODUCTION

**FIG 1:**
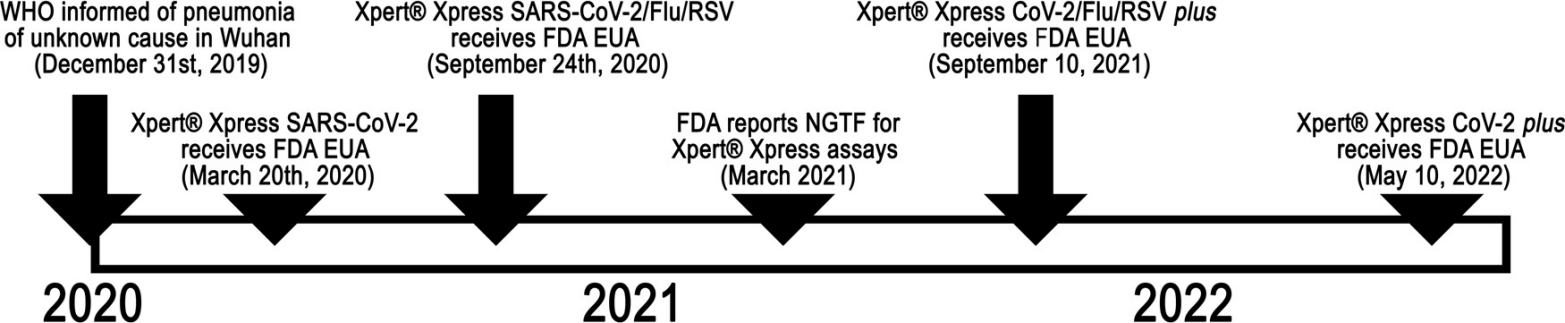
Timeline depicting the evolution of the Xpert Xpress SARS-CoV-2, SARS-CoV-2/Flu/RSV, CoV-2/Flu/RSV-plus, and CoV-2-plus *in vitro* diagnostic (IVD) platforms with U.S. Food and Drug Administration (FDA) emergency use authorization (EUA). WHO, World Health Organization.

Severe acute respiratory syndrome coronavirus 2 (SARS-CoV-2) was first recognized in Wuhan, China, in late 2019, leading to the coronavirus disease 2019 (COVID-19) pandemic. The U.S. Food and Drug Administration (FDA) enabled developers to request emergency use authorization (EUA), which enabled the rapid authorization of *in vitro* diagnostic (IVD) platforms for the detection of SARS-CoV-2. The first FDA EUA IVDs were available to diagnostic laboratories in early 2020, including Xpert Xpress SARS-CoV-2 (1st-generation SARS-CoV-2 Xpress) (Cepheid, Sunnyvale, CA), which received FDA EUA on 20 March 2020. Subsequently, Xpert Xpress SARS-CoV-2/Flu/RSV (1st-generation 4-in-1 Xpress) was developed and received FDA EUA on 24 September 2020 (Fig. 1).

In March 2021, the FDA reported that SARS-CoV-2 with a nucleocapsid (N) gene point mutation may cause N gene target failure (NGTF) for the 1st-generation Xpress tests (1). Synonymous mutations in the N2 probe region (g.29200C>A or g.29200C>T) were identified as the causes of NGTF (2–4). These mutations were uncommon (<2% prevalence in sequenced genomes) and not lineage-defining mutations or specifically associated with variants of concern (VOCs), but the occurrence of target failure was concerning (2). In July 2021, we reported an additional synonymous mutation (g.29197C>T) that was briefly common in northeastern Ohio and also caused NGTF (5). NGTF did not impact the qualitative performance of 1st-generation Xpress tests since the envelope (E) gene target was successfully detected; however, the threat of additional mutations resulting in multiple target failures became a theoretical concern.

During the 2 years following SARS-CoV-2’s emergence, the virus’s RNA genome has evolved. These mutations and polymorphisms can impact the infectiousness and immunogenicity of the virus, but the changes can also impact the performance of IVDs. Mutations can yield reduced sensitivity or complete failure in the detection of genomic regions that are targets of nucleic acid amplification tests (NAATs) (2–11). Most IVD NAATs used to detect SARS-CoV-2 contain redundancy, so if one target fails due to a viral genomic mutation, the qualitative detection of the virus remains intact (7), but some variants have mutations that can cause complete diagnostic failure (1). Specifically, the B.1.1.529 lineage (Omicron) has mutations that can cause the complete failure of a few IVD NAATs that have received FDA EUA (1).

In this article, we describe our experience with postmarket monitoring of the performance of IVDs that target a novel and rapidly evolving virus. We describe the approach used to monitor performance, design and implement assay improvements, and achieve regulatory authorization. We also evaluate the performance of the original and the improved IVDs.

## RESULTS

The *in vitro* performances of the 1st- and 2nd-generation Xpress assays were evaluated with a set of 50 challenge samples. The results of testing 31 samples with synonymous N gene mutations are described in Table 1, and the results of testing 19 samples, including VOCs, without notable N gene mutations are described in Table 2.

**TABLE 1:** Characteristics of samples with notable N gene polymorphisms and results of testing with 1st- and 2nd-generation SARS-CoV-2 Xpert Xpress assays

Sample	Pango lineage	NextStrain clade	WHO VOC designation^*a*^	Relevant N gene mutation	1st-generation SARS-CoV-2 Xpress^*b*^ interpretation	1st-generation SARS-CoV-2 Xpress^*b*^ *C_T_* value		2nd-generation CoV-2 RUO^*c*^ interpretation	2nd-generation CoV-2 RUO^*c*^ *C_T_* value			2nd-generation CoV-2/Flu/RSV-plus Xpress^*b*^ interpretation	2nd-generation CoV-2/Flu/RSV-plus Xpress^*b*^ *C_T_* value for SARS-CoV-2
						E	N2		E	N2	RdRp		
CD001	B.1	20B	NA	g.29197C>T	SARS-CoV-2 presumptive positive	32.0	0.0	SARS-CoV-2 positive	30.6	37.1	32.7	SARS-CoV-2 positive	33.9
CD002	B.1	20B	NA	g.29197C>T	SARS-CoV-2 presumptive positive	34.8	0.0	SARS-CoV-2 positive	31.8	38.3	34.0	SARS-CoV-2 positive	33.8
CD003	B.1	20B	NA	g.29197C>T	SARS-CoV-2 presumptive positive	31.1	0.0	SARS-CoV-2 positive	30.2	36.5	32.6	SARS-CoV-2 positive	34.5
CD004	B.1.1.222	20B	NA	g.29197C>T	SARS-CoV-2 presumptive positive	30.1	0.0	SARS-CoV-2 positive	31.1	37.3	33.5	SARS-CoV-2 positive	31.6
CD005	B.1.1.222	20B	NA	g.29197C>T	SARS-CoV-2 presumptive positive	21.5	0.0	SARS-CoV-2 positive	21.2	27.6	23.1	SARS-CoV-2 positive	22.3
CD006	B.1.1.222	20B	NA	g.29197C>T	SARS-CoV-2 presumptive positive	22.7	0.0	SARS-CoV-2 positive	21.4	28.8	23.3	SARS-CoV-2 positive	23.2
CD007	B.1.1.222	20B	NA	g.29197C>T	SARS-CoV-2 presumptive positive	16.4	0.0	SARS-CoV-2 positive	15.2	23.0	16.8	SARS-CoV-2 positive	17.0
CD008	B.1.1.222	20B	NA	g.29197C>T	SARS-CoV-2 positive	29.0	43.7	SARS-CoV-2 positive	27.3	33.7	29.2	SARS-CoV-2 positive	26.5
CD009	B.1.1.222	20B	NA	g.29197C>T	SARS-CoV-2 presumptive positive	32.0	0.0	SARS-CoV-2 positive	33.8	41.3	38.2	SARS-CoV-2 positive	33.3
CD010	B.1.1.519	20B	NA	g.29197C>T	SARS-CoV-2 presumptive positive	29.6	0.0	SARS-CoV-2 positive	28.4	35.1	30.4	SARS-CoV-2 positive	31.0
CD011	B.1.1.519	20B	NA	g.29197C>T	SARS-CoV-2 presumptive positive	17.5	0.0	SARS-CoV-2 positive	16.4	22.6	18.2	SARS-CoV-2 positive	17.8
CD012	B.1.1.519	20B	NA	g.29197C>T	SARS-CoV-2 presumptive positive	18.4	0.0	SARS-CoV-2 positive	17.7	23.9	20.0	SARS-CoV-2 positive	19.0
CD013	B.1.1.519	20B	NA	g.29197C>T	SARS-CoV-2 presumptive positive	23.7	0.0	SARS-CoV-2 positive	23.1	29.6	25.0	SARS-CoV-2 positive	25.0
CD014	B.1.1.519	20B	NA	g.29197C>T	SARS-CoV-2 presumptive positive	16.8	0.0	SARS-CoV-2 positive	16.6	23.3	18.4	SARS-CoV-2 positive	18.0
CD015	B.1.1.519	20B	NA	g.29197C>T	SARS-CoV-2 presumptive positive	25.7	0.0	SARS-CoV-2 positive	26	32.5	28.1	SARS-CoV-2 positive	27.4
CD016	B.1.1.519	20B	NA	g.29197C>T	SARS-CoV-2 presumptive positive	20.7	0.0	SARS-CoV-2 positive	20.1	26.9	22.1	SARS-CoV-2 positive	21.5
CD017	B.1.1.519	20B	NA	g.29197C>T	SARS-CoV-2 presumptive positive	25.0	0.0	SARS-CoV-2 positive	26	32.9	28.0	SARS-CoV-2 positive	28.1
CD018	B.1.1.519	20B	NA	g.29197C>T	SARS-CoV-2 presumptive positive	29.0	0.0	SARS-CoV-2 positive	30.3	37.6	32.4	SARS-CoV-2 positive	33.1
CD019	B.1.1.519	20B	NA	g.29197C>T	SARS-CoV-2 presumptive positive	16.1	0.0	SARS-CoV-2 positive	15.1	20.8	17.1	SARS-CoV-2 positive	17.1
CD020	B.1.1.519	20B	NA	g.29197C>T	SARS-CoV-2 positive	27.4	42.8	SARS-CoV-2 positive	26.9	33.0	28.9	SARS-CoV-2 positive	28.2
CD021	B.1.1.519	20B	NA	g.29197C>T	SARS-CoV-2 presumptive positive	18.2	0.0	SARS-CoV-2 positive	17.8	24.5	19.4	SARS-CoV-2 positive	19.3
CD022	B.1.1.519	20B	NA	g.29197C>T	SARS-CoV-2 presumptive positive	21.9	0.0	SARS-CoV-2 positive	20.9	27.6	22.5	SARS-CoV-2 positive	22.4
CD023	B.1.1.519	20B	NA	g.29197C>T	SARS-CoV-2 presumptive positive	22.6	0.0	SARS-CoV-2 positive	21.3	27.8	23.1	SARS-CoV-2 positive	23.3
CD024	B.1.1.519	20B	NA	g.29197C>T	SARS-CoV-2 presumptive positive	16.4	0.0	SARS-CoV-2 positive	15.1	21.5	17.0	SARS-CoV-2 positive	18.5
CD032	B.1.1.519	20B	NA	g.29197C>T	SARS-CoV-2 presumptive positive	16.7	0.0	SARS-CoV-2 positive	15.9	22.9	17.5	SARS-CoV-2 positive	17.4
CD033	B.1.1.519	20B	NA	g.29197C>T	SARS-CoV-2 presumptive positive	18.4	0.0	SARS-CoV-2 positive	17.7	24.8	19.1	SARS-CoV-2 positive	19.5
CD034	B.1.1.519	20B	NA	g.29197C>T	SARS-CoV-2 presumptive positive	16.3	0.0	SARS-CoV-2 positive	15.4	22.5	17.2	SARS-CoV-2 positive	17.3
CD035	B.1.1.519	20B	NA	g.29197C>T	SARS-CoV-2 presumptive positive	26.2	0.0	SARS-CoV-2 positive	26.0	32.9	28.0.2	SARS-CoV-2 positive	27.5
CD036	AY.26	21A	Delta	g.29200C>T	SARS-CoV-2 presumptive positive	24.5	0.0	SARS-CoV-2 positive	22.6	26.4	24.6	SARS-CoV-2 positive	24.5
CD037	B.1.1.519	20B	NA	g.29197C>T	SARS-CoV-2 presumptive positive	18.1	0.0	SARS-CoV-2 positive	16.7	22.8	18.7	SARS-CoV-2 positive	19
CD039	B.1.1.519	20B	NA	g.29197C>T	SARS-CoV-2 presumptive positive	27.1	0.0	SARS-CoV-2 positive	27.7	34.9	29.3	SARS-CoV-2 positive	30.1

a NA, not applicable.

b The assay has been issued U.S. Food and Drug Administration emergency use authorization.

c The research use only (RUO) assay has not been authorized as a diagnostic device by the U.S. Food and Drug Administration. This assay is technically equivalent to the Xpress CoV-2-plus test, which received FDA EUA after this testing was performed.

**TABLE 2:** Characteristics of samples with various lineages, including variants of concern, and results of testing with 1st- and 2nd-generation SARS-CoV-2 Xpert Xpress assay

Sample	Pango lineage	NextStrain clade	WHO VOC designation	1st-generation SARS-CoV-2 Xpress^*a*^ interpretation	1st-generation SARS-CoV-2 Xpress^*a*^ *C_T_* value		2nd-generation CoV-2 RUO^*b*^ interpretation	2nd-generation CoV-2 RUO^*b*^ *C_T_* value			2nd-generation CoV-2/Flu/RSV-plus Xpress^*a*^ interpretation	2nd-generation CoV-2/Flu/RSV-plus Xpress a *C_T_* value for SARS-CoV-2
					E	N2		E	N2	RdRp		
CD025	B.1.1.7	20I/501Y.V1	Alpha	SARS-CoV-2 positive	31.6	33.7	SARS-CoV-2 positive	26.3	28.9	28.9	SARS-CoV-2 positive	28.6
CD026	B.1.351	20H/501Y.V2	Beta	SARS-CoV-2 positive	25.3	28.0	SARS-CoV-2 positive	25.2	28.3	26.7	SARS-CoV-2 positive	25.0
CD027	B.1.429	20C	Epsilon	SARS-CoV-2 positive	19.8	22.3	SARS-CoV-2 positive	21.1	23.9	22.6	SARS-CoV-2 positive	21.7
CD028	B.1.525	20A	Eta	SARS-CoV-2 positive	20.4	22.7	SARS-CoV-2 positive	19.8	22.6	21.4	SARS-CoV-2 positive	22.1
CD029	B.1.526	20C	Iota	SARS-CoV-2 positive	20.2	22.8	SARS-CoV-2 positive	19.1	22.2	20.6	SARS-CoV-2 positive	20.2
CD030	B.1.621	20A	Mu	SARS-CoV-2 positive	35.1	37.2	SARS-CoV-2 positive	33.1	36.1	35.5	SARS-CoV-2 positive	34.5
CD031	B.1.617.2	21A	Delta	SARS-CoV-2 positive	26.2	27.6	SARS-CoV-2 positive	22.1	25.9	24.6	SARS-CoV-2 positive	24.7
CD038	B.1.617.2	21A	Delta	SARS-CoV-2 positive	28.3	31.1	SARS-CoV-2 positive	27.3	30.8	29.3	SARS-CoV-2 positive	28.6
CD040	B.1.2	20G	NA^*c*^	SARS-CoV-2 positive	26.6	28.4	SARS-CoV-2 positive	25.6	28.3	27.8	SARS-CoV-2 positive	27
CD041	B.1.529.1	21K	Omicron BA.1	SARS-CoV-2 positive	17.2	19.4	SARS-CoV-2 positive	15.7	19.2	17.5	SARS-CoV-2 positive	17.3
CD042	B.1.529.1	21K	Omicron BA.1	SARS-CoV-2 positive	19.8	22	SARS-CoV-2 positive	18.7	22.1	20.3	SARS-CoV-2 positive	19.5
CD043	B.1.529.1	21K	Omicron BA.1	SARS-CoV-2 positive	17.2	19.7	SARS-CoV-2 positive	15.7	19.5	17.4	SARS-CoV-2 positive	17.3
CD044	B.1.529.1	21K	Omicron BA.1	SARS-CoV-2 positive	18.4	20.6	SARS-CoV-2 positive	17	20.7	18.9	SARS-CoV-2 positive	18.5
CD045	B.1.529.1	21K	Omicron BA.1	SARS-CoV-2 positive	22.8	25.1	SARS-CoV-2 positive	21.9	25.3	23.8	SARS-CoV-2 positive	22.8
CD046	B.1.529.1	21K	Omicron BA.1	SARS-CoV-2 positive	22.2	24.5	SARS-CoV-2 positive	21.2	24.9	23.2	SARS-CoV-2 positive	22
CD047	B.1.529.1	21K	Omicron BA.1	SARS-CoV-2 positive	29.6	32.5	SARS-CoV-2 positive	27.9	31.8	30.1	SARS-CoV-2 positive	29.1
CD048	B.1.529.1	21K	Omicron BA.1	SARS-CoV-2 positive	27.2	28.9	SARS-CoV-2 positive	25.1	28.6	27.3	SARS-CoV-2 positive	27.2
CD049	B.1.529.1	21K	Omicron BA.1	SARS-CoV-2 positive	21.2	23.4	SARS-CoV-2 positive	19.4	22.9	21.4	SARS-CoV-2 positive	20.9
CD050	B.1.529.1	21K	Omicron BA.1	SARS-CoV-2 positive	26.5	28.9	SARS-CoV-2 positive	24.4	27.9	26.7	SARS-CoV-2 positive	26.6

a The assay has been issued U.S. Food and Drug Administration emergency use authorization.

b The research use only (RUO) assay has not been authorized as a diagnostic device by the U.S. Food and Drug Administration. This assay is technically equivalent to the Xpress CoV-2-plus test, which received FDA EUA after this testing was performed.

c NA, not applicable.

The majority of the samples with notable N gene polymorphisms (g.29197C>T or g.29200C>T) had complete NGTF (94%; 29/31) when using the 1st-generation SARS-CoV-2 Xpress test (Table 1), which produced an interpretation of “presumptive positive” according to the FDA EUA IVD instructions for use (IFU). The remaining samples had partial NGTF with detectable but delayed N gene detection (7%; 2/31), which yielded an interpretation of “positive” according to the IFU. The E gene target was detected in all 31 samples with NGTF (100%; 31/31). When testing samples without notable N gene polymorphisms (Table 2), the 1st-generation SARS-CoV-2 Xpress test detected both the N gene and the E gene in all samples (100%; 19/19), which produced an interpretation of positive.

When using the 2nd-generation CoV-2 Xpress research use only (RUO) assay, all three gene targets (N2, E, and RNA-dependent RNA polymerase [RdRp]) were detected in all samples (100%; 50/50). When using the 2nd-generation 4-in-1 Xpress test, all samples, including those with N gene polymorphisms and VOCs, were qualitatively positive for SARS-CoV-2 (100%; 50/50) according to the IFU.

In the NGTF cohort (*n* = 31), there was a statistically significant difference between the threshold cycle Δ*C_T_* values (N2 − E = delta) of the Xpert Xpress SARS-CoV-2 (1st-generation) test (mean = 21.4, standard deviation [SD] = 5.8, and median = 22.3) and the Δ*C_T_* values of the Xpert Xpress CoV-2-plus (2nd-generation CoV-2 Xpress RUO) assay (mean = 6.6, SD = 0.7, and median = 6.5) (*P* < 0.0001). In the VOC cohort (*n* = 19), there was a statistically significant difference between the Δ*C_T_* values of the Xpert Xpress SARS-CoV-2 (1st-generation) test (mean = 2.3, SD = 0.37, and median = 2.3) and those of the Xpert Xpress CoV-2-plus (2nd-generation CoV-2 Xpress RUO) assay (mean = 3.3, SD = 0.4, and median = 3.5) (*P* < 0.0001).

There was a significant difference between the Δ*C_T_* values of the NGTF samples (mean = 21.5, SD = 5.8, and median = 22.3) and those of the VOC samples (mean = 2.3, SD = 0.37, and median = 2.3) with the Xpert Xpress SARS-CoV-2 (1st-generation) test [*t*(14.3) = 48; *P* < 0.0001]. There was also a significant difference between the Δ*C_T_* values of the NGTF samples (mean = 6.5, SD = 5.8, and median = 6.5) and those of the VOC samples (mean = 3.3, SD = 5.8, and median = 3.5) with the Xpert Xpress CoV-2-plus (2nd-generation CoV-2 Xpress RUO) assay [*t*(18.5) = 48; *P* < 0.0001].

## DISCUSSION

In response to the global SARS-CoV-2 pandemic, the U.S. Food and Drug Administration (FDA) enabled developers to request emergency use authorization (EUA), which enabled the rapid authorization of *in vitro* diagnostic (IVD) platforms for the detection of SARS-CoV-2. As a result, many EUA IVD platforms were authorized, including Xpert Xpress SARS-CoV-2 (1st-generation SARS-CoV-2 Xpress) in March 2020 and Xpert Xpress SARS-CoV-2/Flu/RSV (1st-generation 4-in-1 Xpress) in September 2020.

In March 2021, the FDA reported that SARS-CoV-2 with an N gene point mutation may cause N gene target failure (NGTF) for the 1st-generation Xpress tests (1). This communication from the FDA reinforced multiple published reports of NGTF from laboratories throughout the United States (2–11). In the future, there may be the potential for the FDA to encourage the public availability of commercial primer and probe sequences to assist laboratories in monitoring circulating variants and their performance in test methods; however, if the disclosures are not mandated by the FDA for all commercial assays, it would be difficult to encourage competitive commercial entities to release proprietary information.

Mutations in the N2 probe region (g.29200C>A or g.29200C>T) were identified as causes of NGTF, and later, additional synonymous mutations (g.29197C>T) were also reported. Of note, NGTF of the 1st-generation Xpress tests resulted in a qualitative presumptive positive result as the second probe region (E) remained detectable. This is in contrast to a qualitative positive result when both the N2 and E probes were detected. NGTF did not cause false-negative results for SARS-CoV-2; however, growing concern remained over the possibility of future mutations and their impact on the test results. Furthermore, the end-user interpretation of a result of presumptive positive can be challenging or may cause confusion. In our cohort, upon retesting NGTF samples with second-generation assays, no target failures or presumptive positives were identified.

There was a statistically significant difference between the *C_T_* detection of the N2 probes of Xpert Xpress CoV-2-plus (2nd-generation RUO assay) and that of Xpert Xpress SARS-CoV-2 (1st-generation test) regardless of the specimen (NGTF and VOC). There was also a statistically significant difference between *C_T_* detection using NGTF samples and that using VOC samples with both Xpert Xpress SARS-CoV-2 (1st-generation test) and Xpert Xpress CoV-2-plus (2nd-generation RUO assay). The clinical significance of these findings is unclear and reinforces the challenge of attempting to interpret *C_T_* values quantitatively (12, 13). The updated oligonucleotide chemistry in the updated assay prevented NGTF with known mutations, which is a qualitative improvement. The Xpert Xpress SARS-CoV-2 and Xpert Xpress CoV-2-plus tests are authorized for the qualitative detection of SARS-CoV-2 RNA and have no performance claims for real-time reverse transcription-polymerase chain reaction (RT-PCR) *C_T_* values.

The FDA’s EUA of IVD platforms has facilitated the rapid authorization of redesigned IVD platforms like Xpert Xpress CoV-2/Flu/RSV-plus (2nd-generation 4-in-1 Xpress), which received FDA EUA in September 2021. Later, an assay equivalent to the CoV-2 Xpress RUO assay, Xpert Xpress CoV-2-plus, received FDA EUA on 10 May 2022.

In our experience, investigating NGTF findings, reporting SARS-CoV-2 mutations associated with NGTF, having access to global surveillance sequencing data, and the FDA’s EUA pathway worked together to enable the rapid reformulation and authorization of an improved SARS-CoV-2 IVD assay. Monitoring assay performance, evaluating SARS-CoV-2 mutations, and enabling emergency use authorization may also be useful in the future when encountering yet-to-emerge pathogens.

### Conclusion.

Investigating and reporting NAAT target failures are essential for monitoring the postmarket IVD performance of SARS-CoV-2 assays (1–10). The FDA EUA process enables the rapid reformulation and regulatory authorization of improved IVD assays, which can be necessary when encountering a novel and mutating emerging virus such as SARS-CoV-2. In our experience with Xpert Xpress, the reporting of NAAT target failures in first-generation tests, the development of improved second-generation assays, and their subsequent FDA EUA led to improved diagnostic performance during the SARS-CoV-2 pandemic.

## MATERIALS AND METHODS

Using information from the published reports of NGTF and a review of the publicly available genomes in the GISAID (Global Initiative on Sharing All Influenza Data) database, both 1st-generation Xpress tests were redesigned by the manufacturer to mitigate the technical impact of real and potential SARS-CoV-2 mutations. To this end, a third gene target (RNA-dependent RNA polymerase [RdRp]) in the open reading frame 1a/b (ORF1a/b) region was added, and redundant oligonucleotide probes for the N2 target were added to Xpert Xpress CoV-2/Flu/RSV-plus (2nd-generation 4-in-1 Xpress). A similar prototype Xpert Xpress CoV-2-plus (2nd-generation CoV-2 Xpress) research use only (RUO) assay was developed using the same oligonucleotide chemistry, with the exception of the use of different fluorophores for each SARS-CoV-2 gene target. After the completion of the sample testing reported in this study, this RUO assay received regulatory authorization as an FDA EUA IVD assay on 10 May 2022, which is now branded as Xpert Xpress CoV-2-plus.

After receiving institutional review board Cleveland Clinic Institutional Review Board (IRB) Study #: 21-119 approval, 50 unique upper respiratory samples (nasal swabs or nasopharyngeal swabs in 3 mL of normal saline transport medium or universal transport medium) were deidentified, and residual transport medium was tested using the 1st-generation SARS-CoV-2 Xpress test and both 2nd-generation Xpress assays. Thirty-one samples were selected because they had identifiable SARS-CoV-2 polymorphisms associated with NGTF (5). Of the samples associated with NGTF, almost all contained the g.29197C>T polymorphism (97%; 30/31). Nineteen additional samples were chosen because they had genomes that had been prevalent at one time during the pandemic, and these were predominantly samples containing genomes designated variants of concern (VOCs) by the World Health Organization (WHO). Of these 19 samples, half were Omicron (53%; 10/19). One sample (CD0036) had an N gene mutation and was also designated a VOC (Table 1).

Statistical analysis was performed to determine if there was a statistically significant difference in the *C_T_* values of the NGTF samples when run on both the Xpert Xpress SARS-CoV-2 (1st-generation) test and the Xpert Xpress CoV-2-plus (2nd-generation CoV-2 Xpress RUO) assay, and this analysis was repeated using the VOC samples. The samples with NGTF (N2 = 0 [*C_T_*]) were assigned a *C_T_* value of 45, which is the total number of thermocycles performed. Two delta values (N2 − E = delta) were calculated for each sample, one corresponding to the Xpert Xpress SARS-CoV-2 (1st-generation) test and one corresponding to the Xpert Xpress CoV-2-plus (2nd-generation CoV-2 Xpress RUO) assay. The *C_T_* delta values were analyzed using a paired-sample *t* test for the NGTF (Table 1) and VOC (Table 2) cohorts.

Statistical analysis was performed to determine if there was a statistically significant difference between the *C_T_* values of the NGTF samples and those of the VOC samples for each assay. The *C_T_* values generated by the Xpert Xpress SARS-CoV-2 (1st-generation) test were converted to *C_T_* delta values. The delta values of the NGTF samples were compared to those of the VOC samples using an unpaired *t* test. This analysis was repeated for the Xpert Xpress CoV-2-plus (2nd-generation CoV-2 Xpress RUO) assay. All computations were performed using GraphPad Prism (version 8.0.0 for Windows; GraphPad Software, San Diego, CA, USA [www.graphpad.com]). A *P* value of ≤0.01 was considered statistically significant.
